# Dying younger, dying of overdose: gendered and age dimensions of mortality and shelter service access among individuals experiencing homelessness in Toronto, Canada

**DOI:** 10.1186/s12889-026-27460-8

**Published:** 2026-04-22

**Authors:** Farihah Ali, Jordan Mende-Gibson, Nikki Bozinoff, Pamela Leece, Zoë Dodd, Rose A. Schmidt, Jürgen Rehm

**Affiliations:** 1 Institute for Mental Health Policy Research, Centre for Addiction and Mental Health (CAMH), 250 College Street, Toronto, ON M5T 1R8 Canada; 2 Ontario Node, Canadian Research Initiative in Substance Matters (CRISM), 33 Ursula Franklin St, Toronto, ON M5S 2S1 Canada; 3 Division of Social Behavioural Health Sciences, Dalla Lana School of Public Health, University of Toronto, 155 College Street, Toronto, ON M5T 3M6 Canada; 4 Faculty of Applied Health and Community Studies, School of Applied Health, Clinical Research, Sheridan College, 7899 McLaughlin Rd, Brampton, ON L6Y 0P8 Canada; 5 Campbell Family Mental Health Research Institute, Centre for Addiction and Mental Health, 250 College St, Toronto, ON M5T 1R8 Canada; 6 Department of Family and Community Medicine, University of Toronto, 500 University Avenue, 5th floor, Toronto, M5G1V7 Canada; 7 Dalla Lana School of Public Health, University of Toronto, 480 University Ave, Suite 300, Toronto, ON M5G 1V2 Canada; 8 MAP Centre for Urban Health Solutions, Unity Health, Toronto, ON Canada; 9 Department of Internal Medicine, Yale School of Medicine, 356 Cedar St, New Haven, CT 06510 USA; 10 PAHO/WHO Collaborating Centre at Centre for Addiction and Mental Health, 250 College Street, Toronto, ON M5T 1R8 Canada; 11 Department of Psychiatry, Faculty of Medicine, University of Toronto, 250 College Street, Toronto, ON M5T 1R8 Canada; 12 Faculty of Medicine, Institute of Medical Science, University of Toronto, Medical Sciences Building, 1 King’s College Circle, Room 2374, Toronto, ON M5S 1A8 Canada; 13 Center for Interdisciplinary Addiction Research (ZIS), Department of Psychiatry and Psychotherapy, University Medical Center Hamburg- Eppendorf (UKE), Martinistraße 52, Hamburg, 20246 Germany; 14 Program on Substance Abuse & WHO European Region Collaboration Centre, Public Health Agency of Catalonia, 330 Aragó Street, Barcelona, Catalonia 08009 Spain

**Keywords:** Homelessness, Gender, Shelter Usage, Morbidity, Overdose, Harm Reduction, Ontario

## Abstract

**Background:**

Individuals experiencing homelessness in Canada face high morbidity and mortality due to intersecting toxic drug and housing crises. These risks are profoundly gendered; women experiencing homelessness have a median age of death of just 36 years, significantly lower than women in the general population (85 years), and face heightened overdose risk. Despite these inequities, gender-disaggregated analyses across homelessness, shelter systems, and mortality remain limited. This study integrates multiple publicly available datasets to examine how gender and age shape patterns of homelessness, service access, and overdose mortality in Toronto, Ontario.

**Methods:**

We drew on publicly available datasets to examine gendered patterns of homelessness, shelter service use and operations, and mortality in Toronto. Data extracted included: demographics, shelter visits, shelter capacity, service availability, and mortality by cause and location. When possible, data were aggregated by quarter and disaggregated by age and gender. Chi-square tests were performed when possible to assess differences by gender.

**Results:**

Men comprised most people experiencing homelessness (57%), shelter users (58%), and individuals living outdoors (68%). However, women and gender-diverse individuals accounted for a larger share of outdoor deaths occurring at younger ages (< 40 years) compared with men. Although men accounted for most deaths in shelters (78%), there was no significant gender difference in the age of in-shelter mortality (*p* = 0.19). Shelter capacity was structurally gender-imbalanced: there were more men-only beds (37%) than women-only beds (18%). Harm reduction availability was limited across all shelter types and was lowest in women-only shelters (26%). Overdose was leading among women (76% of all deaths) and gender-diverse individuals (80%), compared to 46% of deaths among men. In addition, overdose deaths among women and gender-diverse individuals were significantly more concentrated in younger age groups (< 40 years), compared to men (*p* = 0.024).

**Conclusions:**

While men represent the largest share of the homeless population, women and gender-diverse individuals account for a disproportionate share of overdose and premature deaths, often at younger ages. Limited women-only shelter capacity, scarcity of gender-responsive harm reduction services, and safety concerns in mixed-gender shelters may contribute to women and gender-diverse individuals relying on hidden homelessness or unsheltered settings, where harms are more likely to occur. Addressing these inequities requires expanding women-only and gender-affirming shelter spaces, strengthening trauma-informed and gender-responsive harm reduction, and ensuring staff are trained to provide safe, non-stigmatizing care.

**Supplementary Information:**

The online version contains supplementary material available at 10.1186/s12889-026-27460-8.

## Background

Homelessness remains one of the most urgent public health crises worldwide [1–3]. Recent reports show the highest rates of homelessness in countries across Europe (including the U.K. and France), the U.S., Australia, and Canada [4]. In Canada, Australia, and the U.S., homelessness is marked by high rates of people living in the streets or other public places, compared to countries in Europe where high rates of homelessness are largely among those staying in shelters, marking profound inequities in morbidity, mortality, and access to essential services [1–4]. People experiencing homelessness face dramatically elevated health risks, including higher rates of chronic illness, disability, infectious disease, untreated mental health conditions, and injuries related to structural vulnerability (i.e. poverty, discrimination, unsafe housing) [1–3]. These experiences are also evident in Toronto, Canada’s largest city, where rising housing costs, limited shelter capacity, and inadequate access to supportive housing have intensified exposure to harms for people living in shelters, temporary hotel programs, or outdoors [3]. Homelessness is a structural determinant that shapes exposure to disease, violence, criminalization, and premature death.

The health risks associated with homelessness are substantially magnified for people who use drugs [1]. The ongoing toxic drug crisis, driven by an unpredictable and adulterated illegal, unregulated, street drug supply, has disproportionately affected those without stable housing [5]. People experiencing homelessness often have limited privacy and fewer safe places to use drugs, resulting in rushed and concealed drug use, coupled with diminished access to harm reduction resources such as sterile supplies, supervised consumption services, and drug checking [6]. As a result, people experiencing homelessness face markedly higher rates of overdose, infectious disease transmission, and drug-related injury than their housed counterparts [2, 7]. For example, the U.S. has experienced a viral outbreak in Hepatitis A, disproportionately impacting people who use drugs experiencing homelessness [8]. In Canada, these intersecting crises of homelessness and drug toxicity have transformed the shelter system into a de facto site of overdose response. In Toronto, shelters have taken on this role despite lacking the clinical infrastructure, staffing models, and harm reduction supports typical of dedicated health or harm reduction programs [5, 9]. Staff are routinely expected to respond to overdoses, manage withdrawal-related crises, and support residents’ complex health and social needs, often without adequate training, resources, or clinical infrastructure [9, 10]. In 2024, more than half (55%) of all deaths among people experiencing homelessness in Toronto were due to overdose, compared to 6% who died from cardiovascular diseases and 9% from other chronic or infectious diseases [11]. Furthermore, in 2024, 6% of all opioid overdose deaths in Toronto occurred within shelters, double the provincial rate of 3%, underscoring the central but strained role of the shelter system in the city’s overdose response [12]. The Toronto shelter system operates under immense pressure, simultaneously providing basic survival needs and responding to acute health emergencies for which it was never designed. This pressure has led to shelters to take on responsibilities typically associated with specialized harm reduction programs.

Within this already inequitable landscape, gender and age profoundly shape experiences of overdose risk, access to care, and health outcomes. The mortality gap between people experiencing homelessness and the general population is staggering. In Canada, individuals experiencing homelessness die an estimated 20 to 40 years earlier than their housed counterparts [13]. This disparity is also gendered; [13] in Toronto, the median age at death for women experiencing homelessness dropped to just 36 years in 2024, compared to 85 years among women in the general population [13]. For men, the median age at death was 50 years, compared to 78 years for men in the general population [13]. These figures underscore deep structural inequities shaped by the intersection of the housing crisis, inadequate health and social supports, the ongoing drug toxicity crisis, and broader social inequities, including sexism and gender normativity, that shape gendered patterns of vulnerability [14, 15].

Women experiencing homelessness face unique and overlapping vulnerabilities, including gender-based violence, trauma, stigma related to drug use and motherhood, and heightened exposure to sexual exploitation, that affect their ability to access shelter, safety, and harm reduction services [16]. Prolonged sedation associated with tranquilizer-adulterated opioids might further heighten vulnerability to physical and sexual violence [17, 18]. Despite these well-established inequities, gendered analyses of homelessness, overdose, and mortality remain limited, especially within municipal shelter systems.

Fragmentation of available data makes it challenging to have a clear picture of the gender dynamics of overdose mortality. Toronto publicly reports a wide range of metrics, including shelter occupancy, outdoor homelessness estimates, overdose trends, and mortality surveillance, but these data streams are typically presented in isolation, making it difficult to examine how living conditions, service environments, and causes of death intersect across gender and age. Without integrated analyses, we risk overlooking opportunities to develop interventions that are more equitable and responsive. We address these gaps by integrating publicly available datasets from the City of Toronto, Toronto Public Health, and publicly available shelter websites to provide a descriptive, gender-based analysis of homelessness, shelter service use, and mortality in Toronto. Specifically, we triangulated multiple municipal and public health datasets to examine how living situations (outdoors vs. shelters), service availability within shelters (food/meal programs, clothing/laundry access, housing supports, case management, harm reduction, and peer support), and causes of death intersect with gender, and where possible, age and time period in Toronto, Ontario.

By consolidating fragmented data sources, this paper offers an understanding of how structural conditions, service environments, and gendered inequities shape health outcomes among people experiencing homelessness. Ultimately, this study aims to illuminate the structural and service-related factors that may contribute to gendered patterns of overdose and mortality in Toronto’s homeless population to inform housing, health, and harm reduction interventions globally that are more equitable, responsive, and evidence-driven.

## Methods

### Study design, sources, and indicators

This study used a descriptive analysis of multiple publicly available datasets to examine patterns of homelessness, shelter service operations and use, and mortality in Toronto. Analyses were conducted at the aggregate level, and we integrated datasets to compare patterns across population characteristics, service utilization, and mortality outcomes. Data were extracted and analyzed between August and November 2025. Using multiple sources provided diverse perspectives on the size, composition, and service engagement of people experiencing homelessness, as well as on mortality outcomes. Integrating these datasets enables a comparative gender analysis and offers an understanding of homelessness and mortality dynamics in Toronto than any single data source could provide. Definitions of key terms are presented in Appendix A.

### Data sources and indicators

Table 1 summarizes the datasets and indicators used and how data were extracted and organized.

**Table 1:** Data Source, Indicator, and Extraction

Data Source	Indicator	Description	Gender/Sex Categories Reported	Data Extraction
City of Toronto Street Needs Assessment (20240 [19]	Demographic Profile of People Experiencing Homelessness and the Demographic Profile of the Shelter Population. This includes the number of people experiencing homelessness in Toronto, the length of time experiencing homelessness, living situation (outdoors, city-administered shelters, and Violence Against Women shelter sites), age, and gender.	A point-in-time survey of people experiencing homelessness in Toronto, reporting percentages on gender and age (16–24, 25–44, 45 − 44, 55–64, and 65+) distributions across living situations (outdoors, city-administered shelters, provisionally housed).	Man, woman, gender diverse, trans woman, trans man, and two-spirit.	Percentages extracted directly from public tables. Some indicators (length of time experiencing homelessness, living situation by gender, age distribution) lacked reported n-values and were presented as percentages only.
*Toronto Shelter System Flow* (2024 Q1–2025 Q3) [20]	Shelter Utilization (visits)	Monthly counts of shelter visits in Toronto, disaggregated by age (‘under 16’, 16–24, 25–34, 35–44, 45–54, 55–64, and ‘65 and over’) and gender. Categories include newly identified, returned from housing, and returned to shelter.	Man, woman, gender-diverse/non-binary/two-spirit.	N-values for each age and gender indicator were extracted from the open data source for ‘all populations’ visiting Toronto shelters, which includes individuals experiencing chronic homelessness, refugees, families, youth, single adults, non-refugees, and indigenous individuals. Monthly counts were summed for each yearly quarter (i.e. Q1 - Jan to March), and percentages were calculated.
*Deaths of Shelter Residents* (2024 Q1–2025 Q3) [21]	Shelter Deaths	Monthly counts of Toronto shelter residents who died, disaggregated by gender. Age not reported.	Male, female, non-binary/transgender.	N-values for each gender indicator were extracted from the open data source. Monthly counts were summed for each quarter, and percentages were calculated. Data on age was not available.
*Daily Shelter & Overnight Service Occupancy & Capacity (2024 Q1–2025 Q3) *[22]	Shelter Capacity and Eligibility	Capacity of both city-administered and non-city-run Toronto shelters operational each day, disaggregated by gender designation and youth shelters. Capacity refers to the total number of funded and operational beds available on a given day, not the number of vacant or unoccupied beds. Includes emergency and transitional shelters, as well as related services (24-hour respite, 24-hour drop-in programs, motel/hotel shelters, isolation and recovery sites, warming centres, and ‘out of the cold’ programs)	Men, women, and mixed-adults.	Daily capacities were extracted from the open data source for shelters available to men, women, mixed-adults, and youth. Daily counts were summed up and then averaged for each quarter. A list of shelters operational as of the end of Q3 2025 was also extracted to be used in the data collection process for shelter service availability.
*Toronto Public Health* *Deaths of People Experiencing Homelessness in Toronto* (2024) [11]	Location of Death	The number of deaths among individuals experiencing homelessness was analyzed by location of death (e.g., outdoors, shelter, private residence, hospital or clinic), gender and age group (< 20, 20–39, 40–59, 60+). To assess whether gender and age distributions differed significantly between outdoor deaths and shelter deaths, chi-squared tests were conducted. Statistical testing for other locations was not feasible due to insufficient cell counts.	Female, male, and transgender	N-values on the cause of death and location of death for each age and gender indicator were extracted. Full year data for 2024 was available and aggregated by Toronto Public Health. Quarterly breakdowns for this data were not available.
	Cause of Death	The number of individuals who died by cause (e.g., acute drug toxicity, cardiovascular disease, homicide, suicide, injury), disaggregated by gender and age group. Toronto Public Health received the cause of death by the Office of the Chief Corner, when possible. When this report is not available, Toronto Public Health notes that the cause of death is determined by the service provider or the Toronto Shelter and Support Services.^(21)^ A chi squared test was performed to assess age and gender differences among overdose deaths.		
*Publicly Available Websites (Q3 2025) (see Appendix A)*	Shelter Services	The number of services by gender eligibility. Services include case management, food/meal program, clothing/laundry, harm reduction, peer support, and housing support.		Publicly available information was extracted from the shelter websites. A dataset was then created by the research team, which included information on the shelter name, organization and services listed on the website.

### Data extraction and organization

Data was extracted based on availability for the years 2024 and 2025 to provide a recent picture of homelessness in Toronto. For consistency, when 2025 data was available, only Q1 to Q3 data was collected as full Q4 data was not released at the time of analysis.

Demographic data on people experiencing homelessness were extracted from the *Street Needs Assessment *survey (City of Toronto). Percentages were extracted from the tables and graphs within the assessment. Because this survey does not consistently report ‘n-values’, some indicators are presented as percentages only, reflecting the level of detail publicly available. As a point-in-time survey, the *Street Needs Assessment* does not provide quarterly or monthly breakdowns. Indicators without reported n-values are summarized in Table 1.

Data on shelter visits were extracted monthly from January 2024 to September 2025 from the *Toronto Shelter System Flow* database. Data extracted included total monthly shelter visits (service-use events; repeat entries may be counted multiple times) across ‘all populations’, stratified by age and gender. Monthly totals were aggregated by quarter (Q1 2024- Q3 2025) and organized separately by age group and gender category. Populations included under ‘all populations’ are listed in Table 1.

Data on deaths among shelter residents were extracted monthly from January 2024 to September 2025 from the *Deaths of Shelter Residents* dataset. This data includes the total number of deaths per month, categorized by gender. Age-specific data were unavailable. Monthly counts were aggregated by quarter (Q1 2024 - Q3 2025) and organized by gender by the research team.

Mortality data for people experiencing homelessness were extracted from Toronto Public Health’s *Deaths of People Experiencing Homelessness in Toronto* dashboard. Data included n-values by cause of death, location of death, age group, and gender. The most recent available year (2024) was used, as no 2025 data were available at the time of data extraction. Toronto Public Health categorizes causes of death as: acute drug toxicity, cardiovascular disease, homicide, other disease, pending, suicide, unintentional injury, and unknown. Locations are categorized as: hospital or clinic, other, outdoors, pending, private residence, public building, shelter, and unknown. Acute drug toxicity deaths were further organized by both gender and age group by the research team. Since only annual totals were available, quarterly breakdowns could not be produced.

Information on shelter capacity was compiled from the City of Toronto’s *Daily Shelter & Overnight Service Occupancy & Capacity* dataset. Data were extracted on gender eligibility, indicating whether shelters are open to all genders or specific populations (men-only, women-only, mixed-adults, or youth). The dataset does not specify how transgender or gender-diverse individuals are included in gender-specific shelters, but the City of Toronto notes that all Toronto shelters must adhere to Toronto Shelter Standards, which are currently being revised to better support LGBTQ2S+ individuals [23]. The City of Toronto’s *Daily Shelter & Overnight Service Occupancy & Capacity* reported daily capacities of all shelters operational on each day for mixed-adult, women-only, men-only, and youth shelters. Daily operational bed capacity was aggregated by quarter and disaggregated by shelter designation (men, women, mixed-adult and youth shelters).

For services, the dataset was used to create a list of available shelters as of the end of Q3 2025. Additional data on service availability were extracted from publicly available shelter websites as of October 2025. The list of Toronto shelters and their associated publicly available website is provided in Appendix B.

Due to inconsistencies in the City’s shelter reporting, this study relied on individual shelter websites rather than the City’s generalized descriptions to ensure accuracy in service reporting. For example, the City’s overview states that all shelters provide wraparound supports (e.g., meals, laundry, harm reduction, and case management); however, shelter-level data and websites show considerable variability. Many shelters omit harm reduction or peer support from their listed services.

### Data analysis

Descriptive analyses were conducted across all datasets. Basic frequency counts and percentages were calculated for each indicator and, where possible, disaggregated by gender and age. When monthly data were available, values were aggregated and summarized by quarter (Q1-Q4 2024 and Q1-Q3 2025). Shelter capacity data are reported daily, and each shelter appeared multiple times throughout the quarter for as long as it was operational. To avoid inflating totals due to repeated listings, the daily capacities of all operational shelters were first summed for each day and then averaged across the quarter. This approach accounted for shelters being listed on multiple dates to provide a more accurate estimate of average quarterly capacity. For datasets providing only annual or point-in-time estimates, results were presented as reported by the original source.

Analyses were designed to facilitate comparison across population characteristics (e.g., age, gender), service utilization (e.g., shelter visits, capacity), and mortality outcomes (e.g., cause and location of death). Because publicly available datasets did not include consistent denominators (e.g., unique individuals by gender or age), inferential statistics and rate calculations were not possible. As a result, this analysis does not include capacity-weighted estimates, providing a high-level overview of program availability and not resident-level access. In addition, the *Shelter System Flow* dataset reports counts of shelter visits rather than unique individuals, meaning the same person may be counted multiple times. For this data we conducted descriptive proportional analyses, examining the percentage distribution of demographic characteristics, service utilization patterns and mortality outcomes within and across datasets to identify disparities and temporal trends.

In this study, sex refers to biological attributes (e.g., physiology), whereas gender refers to socially constructed roles, identities, and power relations that shape lived experiences and access to resources [24]. Because the datasets analyzed primarily reported gender identity categories rather than biological sex, this study focuses on gender-based differences in mortality patterns while recognizing that sex-related physiological factors may also influence vulnerability to environmental exposures and health outcomes. A comparative gender analysis was used to examine how mortality patterns, service access, and shelter system characteristics differed across gender groups within the available administrative data. To facilitate comparability across sources, gender categories were harmonized as man, woman, and gender-diverse (transgender, non-binary, two-spirit), following the reporting structures of the City of Toronto and Toronto Public Health. Age categories were analyzed according to each dataset’s reporting schema, as standardized groupings were not possible due to variation across sources.

When possible, chi-squared tests were conducted to assess differences in age and gender distributions across selected indicators. The chi-squared tests evaluated whether observed differences in categorical frequencies deviate from what would be expected by chance [25]. Due to small frequency counts, age categories were collapsed into two groups (< 40 years and ≥ 40 years), and women and gender-diverse individuals were combined into a single category to ensure sufficient sample sizes for analysis. Analyses were conducted in Excel. Because the available datasets do not provide consistent denominators (e.g., the number of individuals at risk within each category), these tests assess differences in the distribution of deaths across groups rather than differences in mortality risk. Table 1 outlines the specific indicators for which chi-squared tests were performed.

### Ethical considerations

All data analyzed were publicly available in aggregate form, with no access to individual-level or identifiable information. This study involved secondary analysis of open datasets and as such, did not require research ethics board approval.

## Findings

### Demographics profile of people experiencing homelessness

As of October 2024, the *Street Needs Assessment* estimated that 15,418 people were experiencing homelessness in Toronto. The majority (83%) were experiencing chronic homelessness: two-thirds (66%) reported being homeless for the entire 12 months preceding the survey, 9% reported being homeless for nine to 11 months, and 8% reported being homeless for six to eight months. The remainder experienced shorter periods of homelessness: four to five months (5%), two to three months (5%), and less than one month (3%).

Overall, men represented the majority (57%) of people experiencing homelessness in Toronto, followed by women (41%) and gender diverse individuals (2%), with an additional 1% declining to state their gender. The average age across all respondents was 41 years. Over half (52%) were between 25 and 44 years old, while 10% were aged 16–24 years, 18% were 45–54 years, 12% were 55–64 years, 5% were 65 years or older, and 3% declined to report their age.

Among youth experiencing homelessness aged 16–24 years, 54% identified as men, 41% as women, and 4% as gender diverse. In the 25–44 age group, men continued to represent a slight majority (52%), with women making up 46% and less than 1% identifying as gender diverse. These gender gaps widened in older age groups; among individuals aged 45–54 years, 63% were men and 36% were women; among those aged 55–64 years, men represented nearly three-quarters (72%), compared to 27% women. Among adults aged 65 and older, men accounted for 68% and women for 32%.

Living situations varied considerably among people experiencing homelessness in Toronto. Of the 15,418 individuals surveyed, the majority (*n =* 12,304; 80%) were staying in city-administered shelters or bridging and triage programs, 10% (*n =* 1,615) were living outdoors, and 10% (*n =* 1,499) were residing in provincially administered VAW shelters. Gender and age distributions differed notably across living settings. Among those living outdoors, men accounted for 68%, women for 30%, and gender diverse individuals for less than 1%. The average age of individuals living outdoors was 41 years, with more than half (53%) between the ages 25–44. The remaining age distribution among those living outdoors was 2% aged 16–24 years, 19% aged 45–54 years, 14% aged 55–64 years, and 6% aged 65+; 4% declined to report their age in the survey.

### Demographic profile of shelter population within city-administered and provincial shelters

Based on the *Street Needs Assessment*, within city-administered shelters, 58% of residents identified as men, 41% as women, and less than 1% as gender diverse, with an additional 2% declining to report their gender. The average age of residents in these settings was 41 years. Half (50%) were between 25 and 44 years old, while the remaining distribution included 11% aged 16–24 years, 18% aged 45–54 years, 12% aged 55–64 years, and 5% aged 65 years or older; 3% declined to report their age. In provincially administered VAW shelters, nearly all residents (99%) identified as women, with fewer than 1% identifying as gender diverse and 1% declining to report their gender. Residents of VAW shelters were younger on average, with a mean age of 36 years. Three-quarters (75%) were between the ages of 25–44 years, followed by 9% aged 16–24 years, 8% aged 45–54 years, and 5% aged 55–64 years; 1% declined to report their age.

### Gender and age-specific shelter services

#### Adult shelter capacity

Based on the City of Toronto’s *Daily Shelter Overnight Service Occupancy and Capacity* open dataset, at the end of Q3 2025, Toronto had 93 adult shelters. Of these, 40% (*n =* 37) were mixed-adult shelters open to all genders, 35% (*n =* 33) were men-only, and 25% (*n =* 23) were women-only.

Mixed-adult shelters consistently held the largest share of total bed capacity. Across Q1 2024 to Q3 2025, they accounted for an average of 45% of beds, corresponding to approximately 2,648 beds during the study period. In Q1 2024, mixed-adult shelters comprised 48% (*n =* 2,964) of all available beds; this proportion declined gradually over the year, reaching 47% (*n =* 2,981) of beds in Q1 2025, and further declining to 43% (*n =* 2,238) in Q3 2025.

Men-only shelters had slightly lower capacity overall, accounting for an average of 37% of beds (approximately 2,162 beds) during the same period. Capacity remained relatively stable between Q1 2024 and Q2 2025, ranging from 35 to 37% (*n =* 2,207 in Q1 2024). By Q3 2025, men-only shelters represented 28% of all beds (*n =* 1,983). Although the number of beds decreased slightly, their share of total capacity increased because mixed-adult shelter capacity declined more substantially during the same period.

**Fig. 1:**
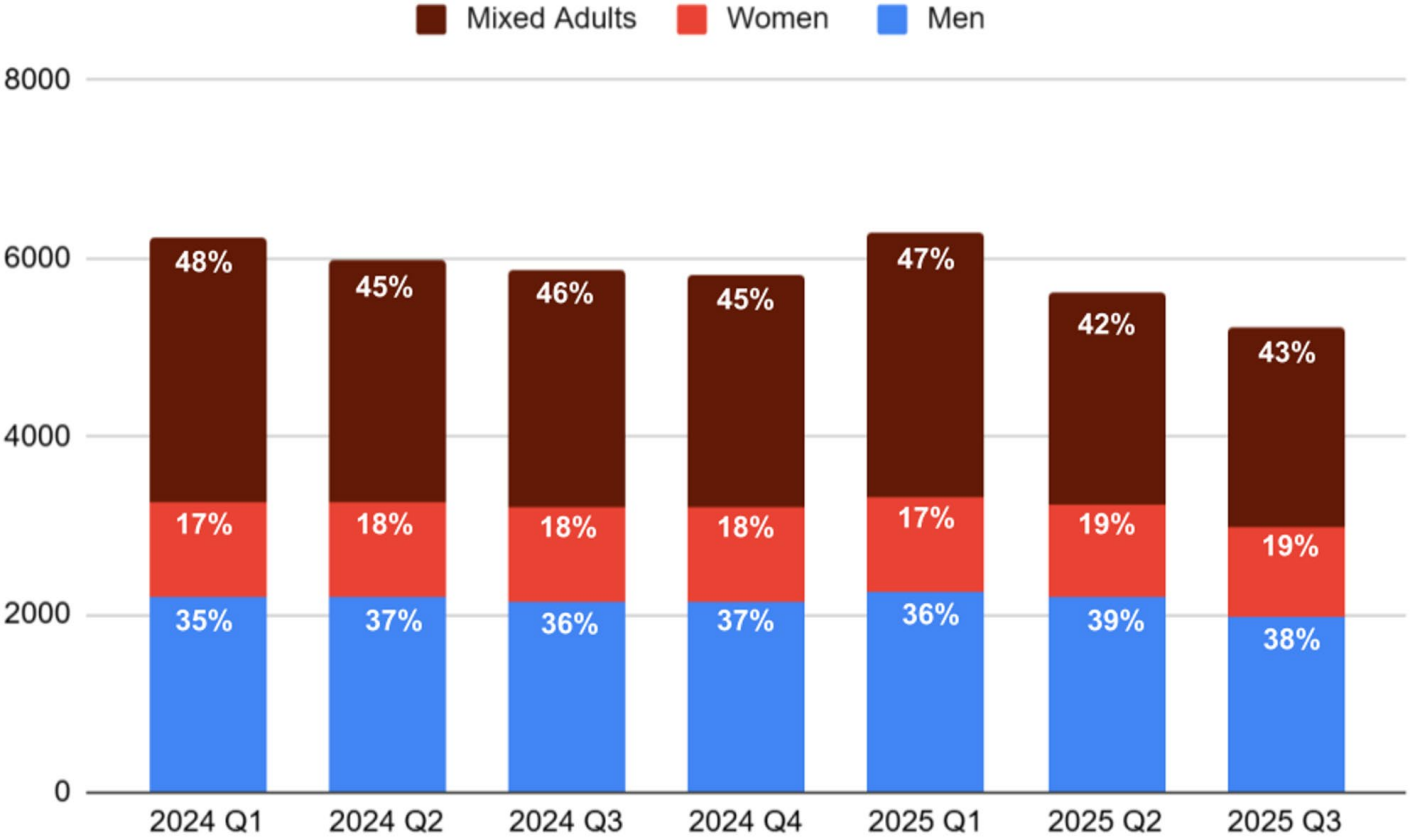
Bed capacity for men, women, and mixed-adults from Q1 2024 to Q3 2025

Women-only shelters were the smallest segment of the system, comprising an average of only 18% of available beds (approximately 1,053) of total beds from Q1 2024 to Q3 2025. Their capacity remained relatively consistent over time, fluctuating narrowly between 17% and 19% each quarter. In contrast to seasonal expansions observed in other shelter types, women-only shelter capacity remained largely unchanged during winter months. See Fig. 1 for an overview of the capacity for men, women, and mixed-adult shelters from Q1 2024 to Q3 2025.

#### Youth shelter capacity

At the end of Q3 2025, Toronto had a total of 25 youth shelters. The majority were mixed-gender (*n =* 17; 68%), including two that specifically served LGBTQ+ youth of any gender (8%). Two shelters serve young women (8%), one serves young men (4%), and the remaining three (12%) did not specify gender eligibility on publicly available materials.

Across all youth shelters, the average capacity from Q1 2024 to Q3 2025 was 699 beds. Capacity levels were relatively stable over time, with modest fluctuations.

**Fig. 2:**
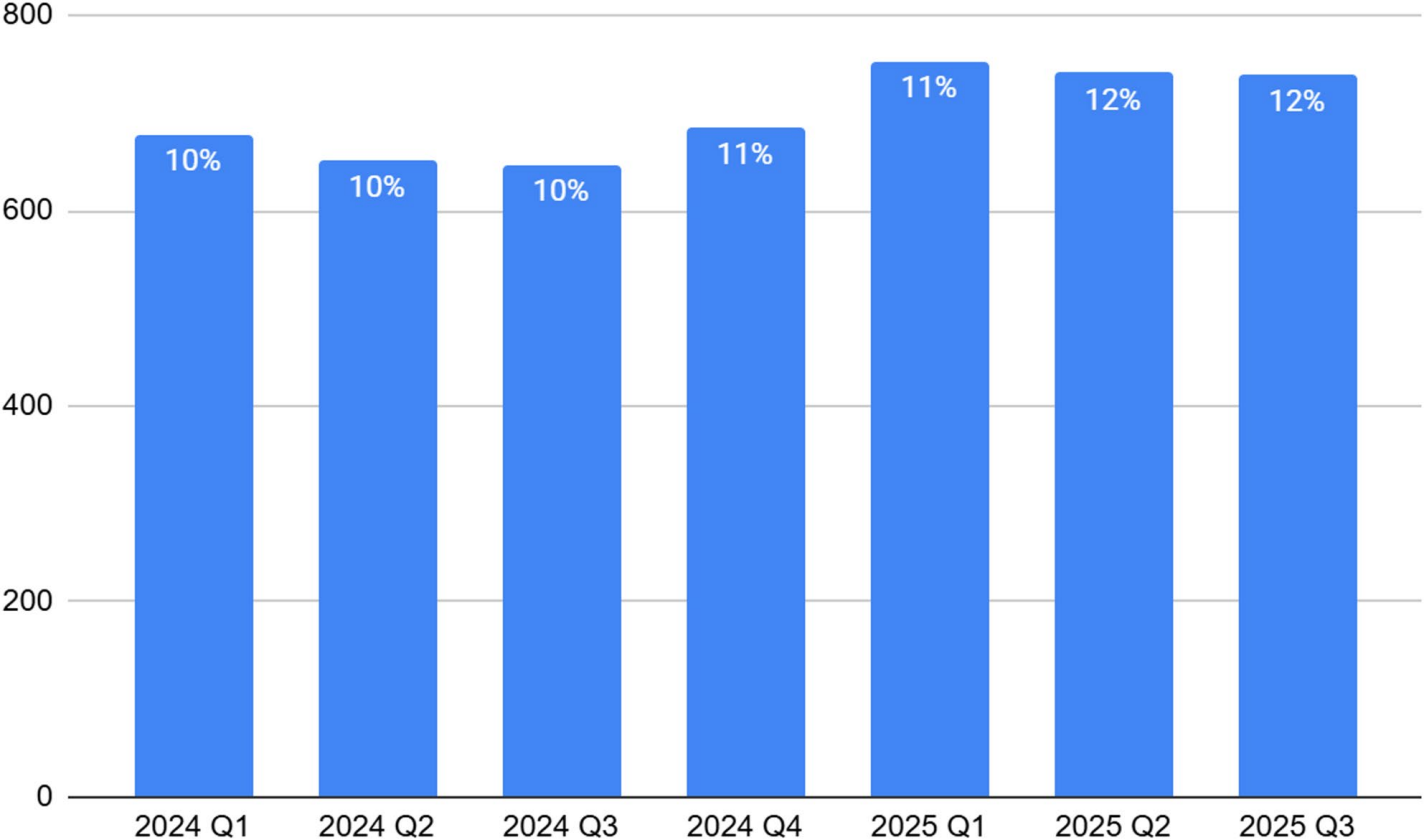
Bed capacity at youth shelters from Q1 2024 to Q3 2025

See Fig. 2 for a complete breakdown of bed capacity at youth shelters from Q1 2024 to Q3 2025.

### Shelter utilization by gender and age

Based on the City of Toronto’s *Shelter System Flow* open dataset, in 2024, there were 128,667 visits to Toronto shelter services. Men accounted for the majority of visits (*n* = 76,250; 59%), followed by women (*n* = 50,923; 40%) and gender-diverse individuals (*n* = 1,494; 1%).

Throughout 2024, men consistently represented the largest proportion of shelter visits. Their share declined slightly mid-year before increasing again in the final quarter, while women’s share increased slightly over the same period. Visits by gender-diverse individuals remained consistently at 1% across all quarters.

**Fig. 3:**
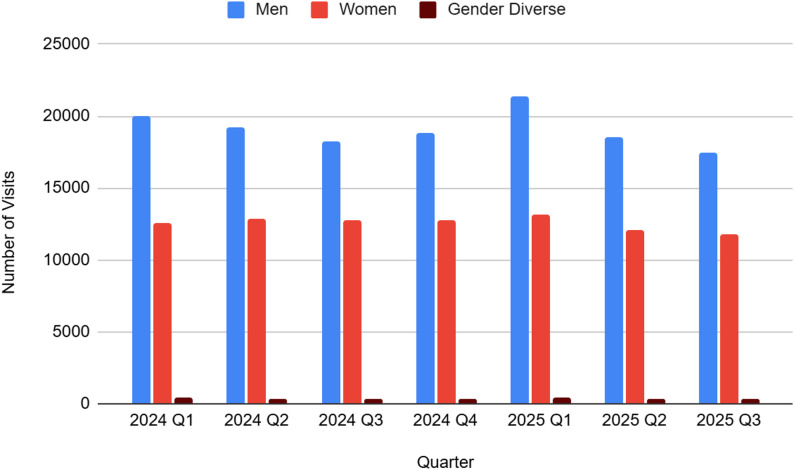
Visits to Toronto shelters by gender for Q1 2024-Q3 2025

This overall pattern of men representing the largest proportion of shelter visits persisted into 2025. However, across the first three quarters, men’s share of shelter visits declined slightly, women’s share increased marginally, and visits by gender-diverse individuals continued to make up 1% of all shelter visits. See Fig. 3 for an overview of visits to Toronto shelters by gender in Q1-Q3 2025.

#### Age distribution

Shelter utilization also varied by age group. In both 2024 and 2025, individuals aged 35–44 years accounted for the highest proportion (23–25%) of visits. Quarterly patterns were generally stable. See Table 2 for the number and percentage of shelter visits by age group for 2024 and Q1-Q3 2025.

**Table 2:** Visits to toronto shelters by age: 2024 and partial (Q1-Q3) 2025

	2024								2025					
	Q1		Q2		Q3		Q4		Q1		Q2		Q3	
	*n*	%	*n*	%	*n*	%	*n*	%	*n*	%	*n*	%	*n*	%
Under 16	3,790	11%	4,159	13%	4,154	13%	4,175	13%	3,902	11%	3,767	12%	3,730	13%
16–24	3,953	12%	3,808	12%	3,803	12%	3,836	12%	4,082	12%	3,772	12%	3,739	13%
25–34	7,756	23%	7,530	23%	7,165	23%	6,899	22%	7,532	22%	6,469	21%	5,947	20%
35–44	7,738	23%	7,551	23%	7,312	23%	7,664	24%	8,721	25%	7,516	24%	6,907	23%
45–54	4,807	15%	4,638	14%	4,402	14%	4,689	15%	5,356	15%	4,660	15%	4,433	15%
55–64	3,337	10%	3,149	14%	3,031	10%	3,164	10%	3,588	10%	3,230	10%	3,182	11%
65+	1,624	5%	1,551	5%	1,474	5%	1,508	5%	1,741	5%	1,626	5%	1,626	5%

### Mortality

#### Deaths of shelter residents

Based on the City of Toronto’s *Deaths of Shelter Residents* dataset, in 2024, 59 individuals residing in Toronto shelters died. Most were men (*n =* 43; 78%), followed by women (*n =* 11; 19%) and gender diverse individuals (*n =* 5; 8%). Deaths decreased steadily across the year, nearly half occurred in Q1 (*n =* 29; 49%), followed by declines in Q2 ( *n =* 14; 24%), Q3 (*n =* 8; 14%), and Q4 (*n =* 8; 14%).

Quarterly patterns showed persistent disparities across genders. In Q1 2024, men accounted for the majority of deaths (*n =* 21; 72%), with women (*n =* 4; 14%) and gender diverse individuals (*n =* 4; 14%) each comprising smaller proportions. In Q2, men again represented the most deaths (*n =* 11; 79%), followed by women (*n =* 2; 14%), and one gender diverse individual (7%). In Q3, deaths were almost exclusively among men (*n =* 7; 88%), with one woman reported (*n =* 1; 13%) and no gender diverse deaths. By Q4, deaths were evenly split between men (*n =* 4; 50%) and women (*n =* 4; 50%), with no gender diverse individuals reported.

In 2025, the absolute number of reported deaths among shelter residents decreased overall compared to the year before. There were 13 deaths in Q1 2025, less than half the number in Q1 2024, with most among men (*n =* 8; 62%) and the remainder among women (*n =* 5; 38%). No gender-diverse deaths were reported. In Q2 2025, deaths increased to 18, slightly higher than Q2 2024, with men again comprising the majority (*n =* 15; 83%), followed by women (*n =* 2; 11%) and one gender diverse individual (*n =* 1; 6%). By Q3 2025, deaths declined once more (*n =* 11), most occurring among men (*n =* 7; 64%) and the remainder among women (*n =* 4; 36%).

**Fig. 4:**
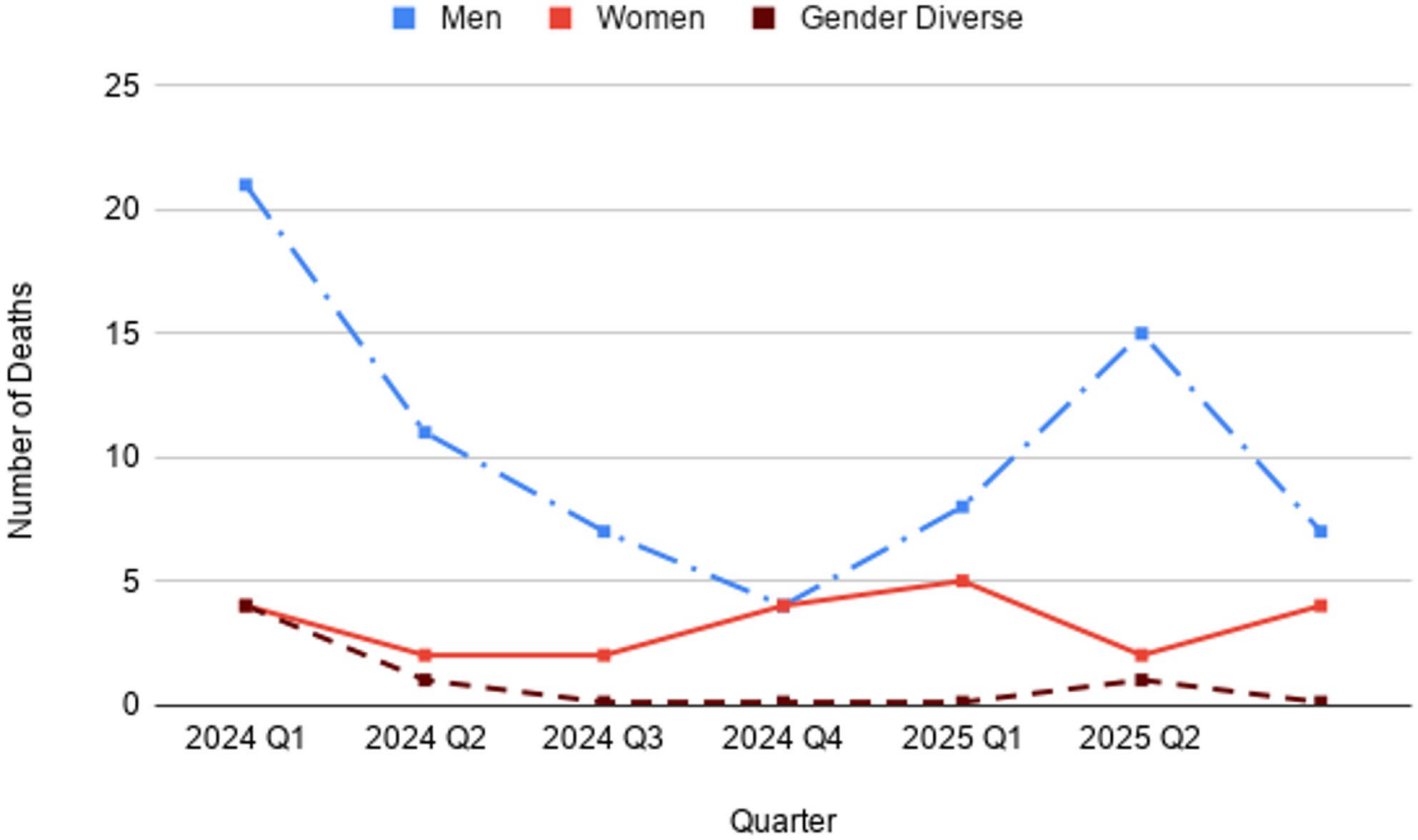
Distribution of deaths of Toronto shelter residents for Q1 2024-Q3 2025

See Fig. 4 for the distribution of deaths among men, women, and gender diverse shelter residents from Q1 2024 to Q3 2025.

Overall, men accounted for a larger share of shelter resident deaths than women; however, these figures represent the distribution of deaths rather than mortality rates, as comparable denominators for shelter populations by gender were not available. Gender diverse individuals are also dying proportionally more in shelters, but small n-values make it difficult to analyze.

See Table 3 for a comparison of shelter visit distributions and deaths among men, women, and gender diverse residents.

**Table 3:** Shelter deaths and visits by gender for Q1 2024 to Q3 2025

	2024								2025					
	Q1		Q2		Q3		Q4		Q1		Q2		Q3	
	*n*	%	*n*	%	*n*	%	*n*	%	*n*	%	*n*	%	*n*	%
Men														
Deaths	21	71%	11	79%	7	88%	4	50%	8	62%	15	83%	7	64%
Visits	19,998	61%	19,177	59%	18,276	58%	18,799	59%	21,327	61%	18,578	60%	17,448	59%
Women														
Deaths	4	14%	2	14%	2	13%	4	50%	5	38%	2	11%	4	36%
Visits	12,591	38%	12,847	40%	12,717	41%	12,768	40%	13,161	38%	12,086	39%	11,749	40%
Gender Diverse Individuals														
Deaths	4	14%	1	7%	0	0%	0	0%	0	0%	1	6%	0	0%
Visits	416	1%	362	1%	348	1%	368	1%	434	1%	376	1%	367	1%

### Deaths among people experiencing homelessness in Toronto

Based on Toronto Public Health’s *Deaths of People Experiencing Homelessness* dashboard, in 2024, 215 deaths were reported among people experiencing homelessness. The majority were men (*n =* 163; 76%), followed by women (*n =* 46; 21%) and gender diverse individuals (*n =* 5; 2%).

#### Location and age distribution of death

Of the 215 deaths among homeless people, nearly one-third (*n =* 69; 32%) occurred outdoors, 20% (*n =* 42) occurred within shelters, 17% (*n =* 37) occurred in private residences, and 13% (*n =* 29) occurred in hospitals or clinics.

The remaining deaths occurred in public buildings (*n =* 19; 9%), unknown locations (*n* = 11; 5%), ‘other’ locations (*n =* 7; 3%), or were pending classification (*n =* 1; <1%). Among these locations, the majority of deaths again clustered among individuals aged 20–59 years, with very few under age 20 years.

#### Gender and age differences in location of death

Among women experiencing homelessness, one-third (*n =* 15; 33%) of deaths occurred outdoors, 22% (*n =* 10) in shelters, 17% (*n =* 8) in private residences, and 11% (*n =* 5) in hospitals or clinics. The remaining deaths (*n =* 8; 17%) occurred in public buildings, and ‘other’ or ‘unknown’ locations. Outdoor deaths and deaths which occurred in private residences were concentrated among younger women while shelter deaths were distributed more evenly across age groups. The majority of deaths in hospitals or clinics were concentrated among older age groups.

Among men, deaths were more evenly distributed across settings. Outdoor deaths represented 32% (*n =* 52) of fatalities, followed by 18% (*n =* 29) in shelters and private residences, and 14% (*n =* 23) in hospitals or clinics, where middle-aged and older men accounted for most deaths. The remaining deaths (*n* = 30; 18%) occurred in public buildings, in ‘other’ or ‘unknown’ locations, or were pending at the time of data collection.

Among gender diverse individuals, most deaths occurred in shelters (*n =* 3; 60%), followed by outdoors (*n =* 1; 20%) and in hospitals or clinics (*n =* 1; 20%). Shelter deaths primarily involved individuals aged 40–59 years.

See Appendix C for a complete breakdown of the location and age of death among men, women, and gender diverse people experiencing homelessness in 2024.

Outdoor location of death was significantly different across genders and age when assessed by a chi-square test (Appendix D, Table 1). This analysis suggests that more women and gender diverse individuals are dying outdoors at younger ages (< 40 years) compared to men (*p* = 0.00002). There was no significant difference in age of mortality by gender for shelter deaths (*p* = 0.19) (Appendix D, Table 2).

### Cause of death

**Fig. 5:**
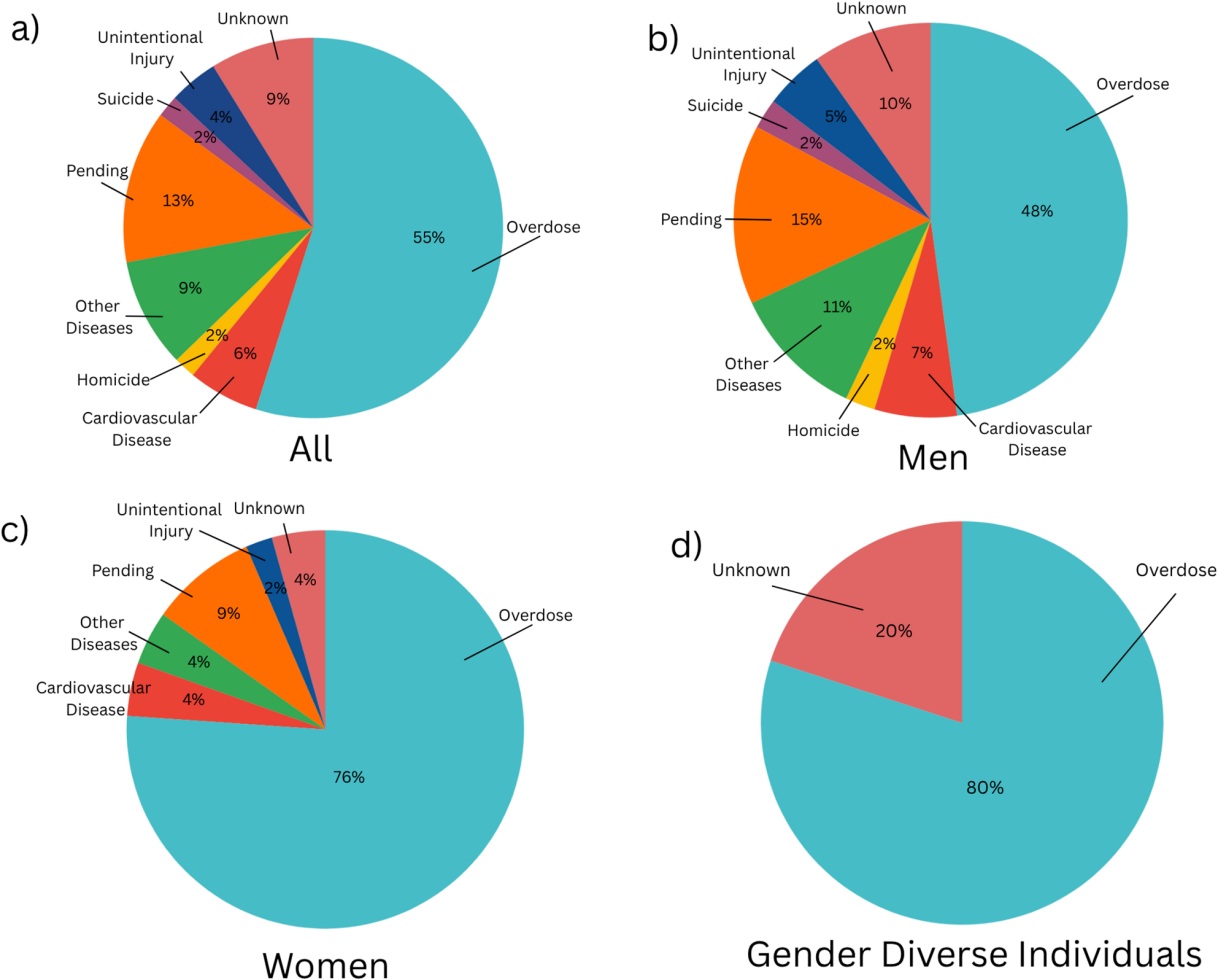
**a**) Cause of death among all genders (men, women, and gender diverse individuals); **b**) cause of deathamong men; **c**) cause of death among women; **d**) cause of death among gender diverse individuals

Just over half of all deaths (*n =* 118; 55%) among people experiencing homelessness during this study period were attributed to overdoses. There were striking differences when disaggregated by gender, with women disproportionately affected. More than three-quarters of all deaths among women (*n =* 35; 76%) were due to overdose. Among gender diverse individuals, the proportion was similarly high, with 80% (*n =* 4) linked to overdose and the remaining death (*n =* 1; 20%) classified as ‘unknown’. In contrast, fewer than half of all deaths among men (*n =* 78 deaths; 48%) were attributed to overdose. See Fig. 5 for a breakdown of cause of death by gender for 2024.

#### Age distribution of overdose deaths

Among men who died of overdose, nearly half (*n =* 36; 46%) were aged 40–59 years, 40% (*n =* 31) were aged 20–39 years, 12% (*n =* 9) were 60 years or older, and 4% (*n =* 2) were of unknown age.

Among women, overdose deaths were concentrated in younger age groups: 60% (*n =* 21) were aged 20–39 years, 29% (*n =* 10) were aged 40–59 years, 6% (*n =* 2) were under 20 years, 3% (*n =* 1) were 60 years or older, and 3% (*n =* 1) were of unknown age.

Among gender diverse individuals, most (*n =* 3;75%) were aged 40–59, and the remainder (*n =* 1; 25%) were aged 20–39. No overdose deaths among gender diverse individuals occurred among those under 20 years, 60 years or older, or of unknown age.

Age of overdose mortality was significantly different between genders when assessed by a chi-square test (Appendix D, Table 3). This analysis suggests that more women and gender diverse individuals are dying of overdose at younger ages (< 40 years) compared to men (*p* = 0.024).

**Fig. 6:**
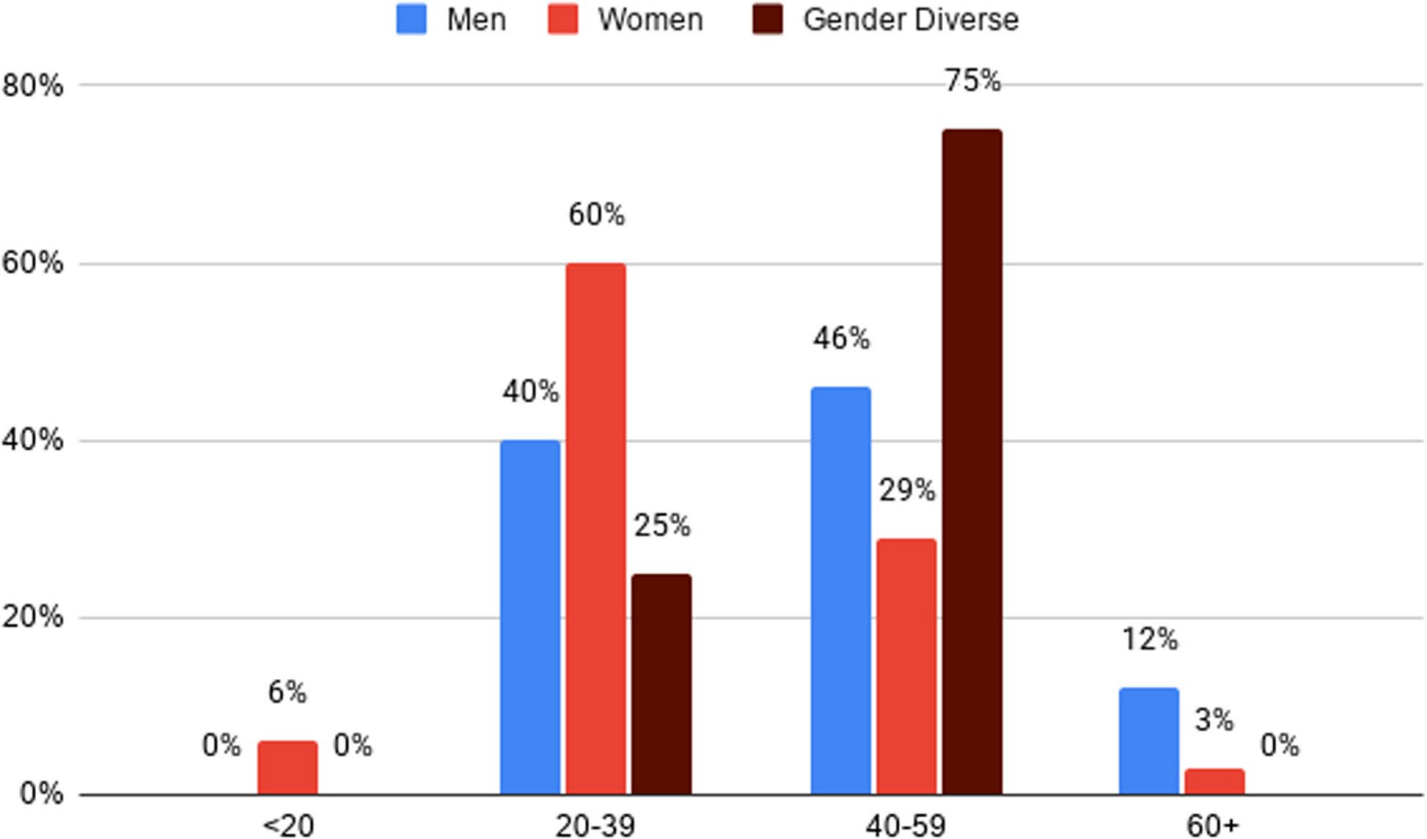
Overdose deaths by age in 2024

See Fig. 6 for a detailed breakdown of overdose deaths by age and gender.

### Shelter service provision and availability by gender and age

#### Adult shelter service provision

Based on the publicly available shelter listings, service availability varied considerably across Toronto’s adult shelters. Most shelters reported offering housing supports (*n =* 71; 76%). Other commonly provided services included food or meal programs (*n* = 59; 63%), case management (*n =* 56; 60%) and clothing or laundry access (*n =* 41; 44%). Specialized supports such as harm reduction services (*n =* 32; 34%) and peer support services (*n* = 8; 9%) were less commonly reported. A small subset of shelters (*n =* 9; 10%) did not specify their available services or did not have publicly available program information (6% of men-only, 11% of mixed-adult, 13% of women-only).

#### Adult shelter service availability by gender designation

Service availability varied modestly across men’s, women’s, and mixed-adult shelters. Housing supports were widely available across shelter types, reported in 85% of men’s shelters (*n =* 28), 83% (*n* = 19) of women’s shelters and 65% (*n* = 24) of mixed-adult shelters. There was some variation across shelter types for food or meal programs and case management provision. Food or meal programs were reported in 74% (*n* = 17) of women’s shelters, 61% (*n* = 20) of men’s shelters and 59% (*n* = 22) of mixed-adult shelters. Case management was reported in 65% (*n* = 15) of women’s shelters, 61% (*n* = 20) of men’s shelters, and 57% (*n* = 21) of mixed-adult shelters. Harm reduction services were less frequently available overall and varied by shelter types, reported in 41% (*n* = 15) of mixed- adult shelters, 33% (*n* = 11) of men’s shelters, and 26% (*n* = 6) of *women’s* shelters. Clothing or laundry access also varied, reported in 57% (*n* = 13) of women’s shelters, 42% (*n* = 14) of men’s shelters, and 38% (*n* = 14) of mixed-adult shelters.

Peer support programs were the least commonly available service across all shelter types, offered by only two men’s shelters (6%), four women’s shelters (17%), and two mixed-adult shelters (5%).

**Fig. 7:**
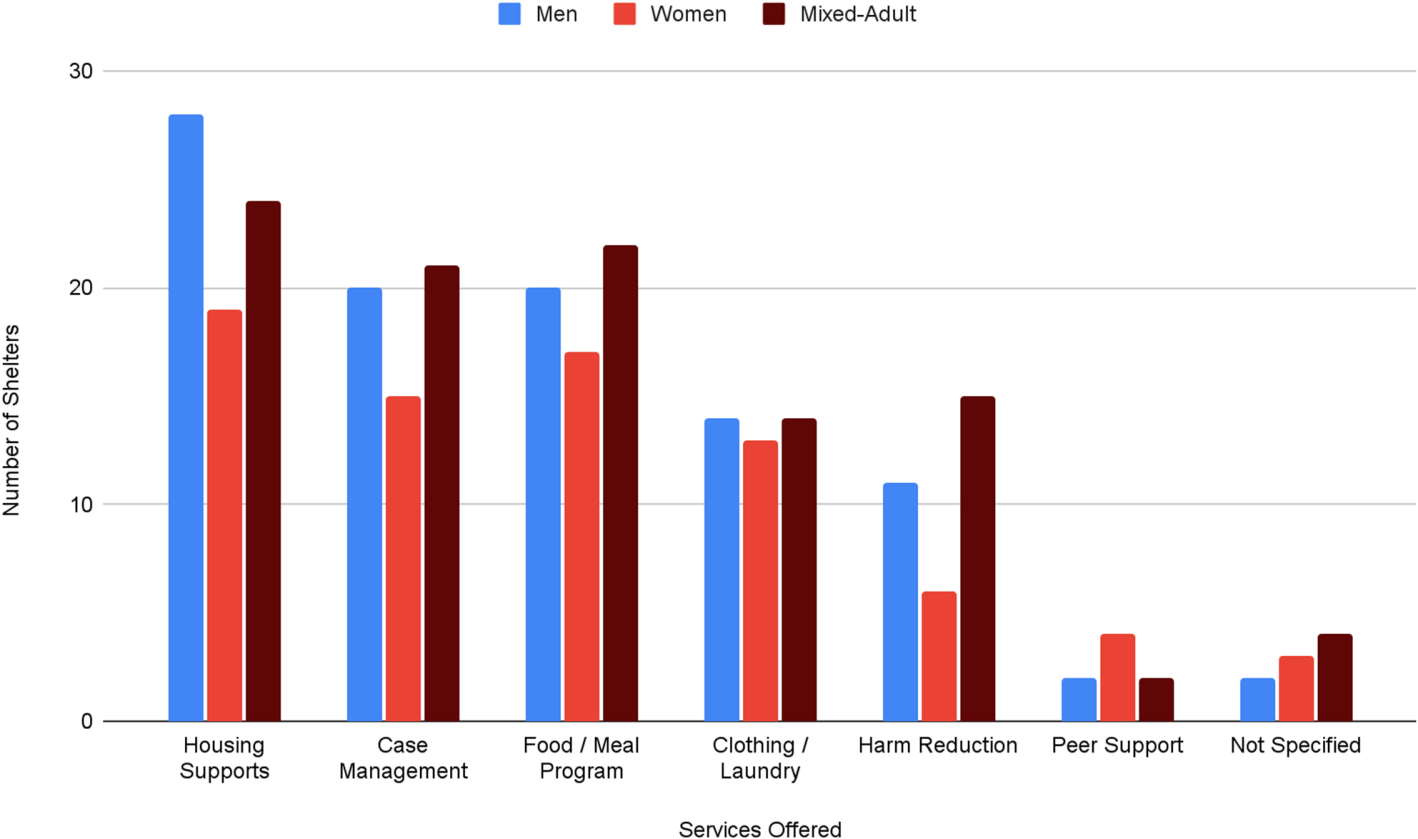
Services available at Toronto shelters as of Q3 2025

See Fig. 7 for a breakdown of services by gender designation.

#### Youth shelter service provision

Service provision across Toronto’s youth shelters was generally limited. A majority offered housing supports (*n =* 15; 60%), while just under half offered food or meal programs (*n =* 12; 48%). Fewer youth shelters offered case management (*n =* 10; 40%), clothing or laundry services (*n* = 7; 28%), or harm reduction services (*n =* 5; 20%). Peer support programs were not available services in any youth shelter (0%). Notably, more than one-quarter (*n =* 7; 28%) did not specify available services or lacked a publicly accessible website.

Service availability also varied considerably by gender designation. Gender-inclusive youth shelters offered housing supports (*n =* 12; 71%), food or meal programs (*n* = 10; 59%), and case management (*n =* 10; 59%). Clothing or laundry services (*n* = 5; 29%) and harm reduction services (*n =* 3; 18%) were less frequently available. Among LGBTQ+ gender-inclusive youth shelters (*n =* 2), one (50%) offered housing supports case management, and food or meal programs, while neither shelter (*n =* 0; 0%) offered clothing or laundry access, harm reduction, or peer support. One program (50%) did not specify its service offerings. Both women-only and youth shelters (*n =* 2) provided housing supports (100%), clothing or laundry services (100%), and food or meal programs (100%). Only one offered case management (*n =* 1; 50%), and neither provided harm reduction services (0%). The sole men-only youth shelter offered housing support (100%) only, without services such as meals, case management, or harm reduction.

## Discussion

Taken together, our findings show that homelessness in Toronto and overdose risk among this population reflect gendered social conditions and structural inequities that shape exposure to risk environments and access to services. Although men make up the largest share of people experiencing homelessness and the majority of shelter users, women and gender-diverse individuals may experience heightened vulnerability across every stage of the homelessness and overdose continuum, from exposure to unsheltered environments, to service access, to cause and age of death [15, 26]. These inequities reflect a misalignment between who is most at risk and how the shelter and harm reduction systems are designed.

Our analysis demonstrates that men account for most individuals experiencing homelessness, shelter visits, and deaths in absolute terms. However, women and gender-diverse individuals face a disproportionate burden of overdose mortality, and at younger ages. Overdose was the leading cause of death for women and gender-diverse individuals, comprising more than three-quarters of deaths in both groups, compared with fewer than half among men. Overdose deaths among women were also concentrated among younger age groups, particularly those aged 20–39 years, despite individuals in this age range representing a relatively small share of the overall homeless population. This pattern aligns with broader evidence that young women who use drugs face heightened overdose risk even without higher prevalence of use, due to intersecting exposures to gender-based violence, trauma, stigma, and inadequate access to gender-responsive services [14, 15, 27, 28].

Further compounding women’s overdose risk is a lack of women-only shelters. Despite comprising over 40% of people experiencing homelessness, only one quarter of Toronto’s adult shelters serve women exclusively. Persistent under-provision of women’s services is a structural inequity that may limit women’s access to safe indoor spaces. Limitations in women’s services are not unique to Toronto. Decades of international research has highlighted limited services for women, particularly young women, experiencing homelessness, which, coupled with experiences of stigma, creates barriers to accessing women’s services even when they are available [15, 29]. The scarcity of dedicated shelter beds might help explain why younger women were overrepresented in outdoor deaths despite being a minority of the outdoor homeless population (comprising only 30% compared to men who comprise 68% of individuals living outdoors). These findings suggest that conditions faced by those living outdoors may be particularly dangerous for younger women, who in our analysis died disproportionately at younger ages in outdoor settings.

Living outdoors during extreme temperatures carries severe health risks, particularly in countries such as Canada, Northern U.S., and Europe, where extreme temperatures in winter and summer months put individuals at risk of frostbite, hypothermia, dehydration, or heat stroke [30]. These health risks may also reflect sex-related physiological differences [31]. Research has consistently shown that women are more sensitive to colder temperatures and take longer to recover from periods of extreme cold [31]. This is due to women typically having a lower metabolic rate which reduces heat production, causing their extremities (hands, feet, face) to receive less blood flow compared to men [31]. During the winter months, the City of Toronto opens additional emergency shelter beds, warming centre spaces when temperature drops to -five Celsius, and “surge” spaces for extreme cold alerts below − 15 Celsius, [32] which may be reflected in our findings showing higher capacities for mixed-adult shelters in Q1 of both 2024 and 2025. While the City’s emergency measures provide temporary reprieve to the dangers of living outdoors in extreme cold weather, they fail to account for the cold-related harms when the weather is above these temperatures, [33] and the 2% of individuals who remain unsheltered year-round.

Temporary expansions in shelter capacity during colder months act primarily as crisis management tools, reinforcing what scholars describe as “reactive homelessness policy,” in which governments respond to visible crises (e.g., extreme cold alerts, encampment deaths), while neglecting the structural drivers, poverty, restrictive income supports, lack of affordable housing, and systemic discrimination that perpetuate cycling between indoor and outdoor environments or produce homelessness [34]. Similar emergency response plans can be seen across the U.S. and Europe, providing temporary shelter expansions during extreme temperatures [35–38]. However, this type of reactive response disproportionately impacts marginalized populations negatively, particularly women, gender- diverse individuals, and youth, because they fail to address the structural and systematic factors that drive inequities [33, 39–42]. The Toronto Auditor has highlighted how temporary expansions were often inadequate, stating that the majority of individuals using winter respite sites or warming centres were either experiencing chronic homelessness or identified as refugees who often require additional supports (e.g., employment or housing supports), which temporary shelters do not often provide [43]. Studies across Canadian and U.S. cities have also demonstrated the harms of an “institutional circuit” or “shelter churn,” where individuals remain unstably housed and move repeatedly between shelters, hospitals, police custody, and outdoor settings due to the absence of permanent housing options [43]. People who use emergency spaces or warming centres often experience “shelter churn,” putting them at risk of increased morbidity, exacerbated mental health conditions, and heightened overdose risk [42–44].

For women and gender-diverse people in particular, temporary expansions often fail to translate into increased shelter accessibility. Warming centres are typically mixed-gender and designed for short stays. Our findings showed no increase in capacity for women-only shelters during the winter months, meaning that the only increased capacity for shelter options for women living outdoors during this time would be to attend mixed-gendered warming centres, often perceived as unsafe [3, 45]. As a result, even when temporary spaces open, women may still opt to remain outdoors or in precarious, “hidden homelessness” arrangements, such as couch surfing, staying with friends or unsafe partners, or exchanging sex for housing, placing them at far greater risk of preventable harm [14, 27, 46, 47].

Inequities in the provision of shelter services are compounded by the limited availability of harm reduction supports within women’s shelters. In our analysis, harm reduction services were largely concentrated in mixed-adult shelters, while women-only shelters offered few or none (41% at mixed-adult shelters compared to 26% at women-only shelters). However, research consistently shows that women avoid mixed-adult shelters due to safety concerns, past trauma, fear of violence, and retraumatization, often relying instead on “hidden homelessness” arrangements [3, 27, 46, 47] Further, women who use drugs often consume drugs in hidden or solitary settings to avoid stigma, child protection involvement, or partner violence [48, 49]. Concealed drug use increases the likelihood of unwitnessed overdoses and reduces opportunities for timely intervention, [50, 51] highlighting the need for harm reduction services in women-only shelters. These risks are exacerbated among women of color. For instance, black and Indigenous mothers are more likely to be drug tested during pregnancy, investigated for maltreatment, and have their child placed in out-of-home care, which can increase surveillance and fear of service involvement [50, 52].

The majority of Toronto’s shelter and harm reduction systems are formally gender-neutral, yet the patterns observed in our analysis suggest unequal mortality patterns and service accessibility across gender groups. Our findings show that gender-diverse individuals had a high concentration of deaths in shelters yet comprised a very small share of total shelter visits. This underscores how institutional spaces intended to provide safety can also become sites of exclusion and harm. Providing trauma-informed care is an essential step in addressing these unequal outcomes. Trauma-informed care acknowledges that many individuals coming into the shelter system have past experiences of violence and trauma that can impact their service engagement [53, 54]. Trauma-informed services are delivered in ways that prioritize safety and trustworthiness, with the aim of preventing shelter spaces from becoming retraumatizing [55]. While both the City of Toronto [56] and Canada’s national shelter standards [57] require implementing a trauma-informed approach, many individuals have continued to report feeling unsafe and having experiences of violence within these settings [58]. For example, safety report of Toronto shelters highlighted that individuals most at risk of violence in shelter settings include individuals who identify as transgender or non-binary, people who use drugs, people of colour, and women [58] The report further stated that staff felt the options for trauma-informed training were often inadequate, and that high rates of staff turnover meant that Toronto shelters often lack sufficiently trained staff [58]. Again, this is not unique to Toronto. For instance, one study in the U.K. found that transgender people experience high incidents of trauma and violence in shelter settings [3]. The study went on to explain how shelter staff normalized this violence and would simply relocate transgender individuals to alternative spaces instead of addressing transphobic behavior [3]. Further, women and gender-diverse individuals are more likely to experience gender-based violence compared to men, which is exacerbated when drug use is involved [59]. However, women and gender-diverse individuals often use drugs to cope in response to trauma, stigma, and violence, which can perpetrate a cycle of trauma, violence, and substance use [59, 60].

The limited availability of women-only beds, absence of trauma-informed shelter design, and scarcity of gender-responsive harm reduction services may contribute to service environments that are less accessible or acceptable to women and gender-diverse individuals who are at heightened risk of fatal overdose [3, 15, 57, 59]. This service gap mirrors broader inequities within the harm reduction infrastructure globally, where few supervised consumption services (SCSs) or harm reduction programs are explicitly designed for women, and even fewer offer trauma-informed, gender-responsive care [9, 61]. Although some gender-specific initiatives have emerged, such as SisterSpace, [62, 63] a women-only overdose prevention site established in Vancouver, Ontario, these programs remain rare and unevenly distributed. Together, the lack of gender-specific services may contribute to a system that is gender-neutral, and therefore appears inclusive in design but may inadequately meet the needs of those at greatest risk of mortality. In Ontario, policy restrictions such as the Community Care and Recovery Act’s (2024) prohibition on SCSs near schools and its ban on new exemptions for shelter-based SCSs, [64] further threaten to eliminate the few harm reduction supports accessible to women and gender-diverse people. This is likely to result in unsheltered women and gender-diverse individuals being forced into isolated or hidden substance use, heightening risks of overdose, and mortality [51]. Addressing gendered inequities will require intentional investment in women and gender diverse-specific programming, peer-led harm reduction, and trauma-informed shelter environments. Shelter sites must also provide training for frontline staff in trauma- and gender-informed care, alongside integrated supports for survivors of violence and grief supports for those who witness overdoses, as many shelters have become de facto SCS when formal harm reduction services are not being provided [57, 58, 65].

Addressing gendered disparities among people experiencing homelessness will require more than incremental expansion of existing services but demands structural redesign. Increased women-only and gender-diverse shelter capacity, trauma- and gender-informed environments, peer-led harm reduction programs, and protections that ensure safe consumption is possible indoors. Research has shown that peer-led harm reduction programs in shelter settings can make people who use drugs feel safer, [66] reducing the feeling of needing to rush injection behaviour, [67] which subsequently could reduce their risk of overdose. However, addressing downstream interventions alone will remain insufficient to meaningfully reduce mortality among women and gender-diverse people experiencing homelessness, and it is important to also work to address upstream determinants of health such as structural racism, gender-based violence, adequate funding for shelter services, and punitive drug policies.

### Limitations

This study draws on multiple publicly available datasets that differ in scope, reporting practices and temporal granularity, which introduces several methodological limitations.

First, reporting timeframes were not consistent across datasets, limiting our ability to conduct longitudinal analyses. The *Street Needs Assessment* is a point-in-time survey capturing conditions on a single day, whereas Toronto Public Health’s *Deaths of People Experiencing Homelessness in Toronto* reports annual mortality totals without monthly or quarterly breakdowns. Consequently, we were unable to examine finer temporal trends in mortality or compare changes over time across datasets. Additionally, 2025 data were available for some sources (e.g., Shelter System Flow) but not for others (e.g., Toronto Public Health’s mortality data and the *Street Needs Assessment)*, resulting in incomplete coverage for that year.

Second, cause-of-death data contained missing or pending classifications at the time of analysis. In Toronto Public Health’s mortality dataset, approximately 15% of deaths were categorized as “pending” at the time of analysis, which may underestimate the true burden of overdose deaths in 2024. Minor inconsistencies between aggregated and disaggregated counts were also observed (e.g., totals for overdose deaths among individuals aged 20–39 years differed slightly when summed by gender), likely reflecting pending classifications or reporting discrepancies.

Third, gender categories were inconsistently defined across datasets and lacked sufficient detail to fully examine gender diversity across the shelter system. Shelter datasets identify programs as “men-only,” “women-only,” or “mixed-adults” without clarifying whether transgender or gender-diverse individuals are included or excluded in gender-specific shelters. While Toronto shelter standards are currently being revised to better support LGBTQ2S+ individuals, these revisions were not captured during our data collection period. Combined with the structural ambiguity of “men-only,” “women-only,” and “mixed-adult” shelters prevents accurate interpretation of service accessibility for transgender and gender-diverse residents. Additionally, due to small cell counts, women and gender-diverse individuals were combined in some analyses, which may obscure distinct experiences and risks faced by transgender and gender-diverse people. Small sample sizes also mean that minor changes in absolute counts can appear as relatively large proportional differences across time or categories, which should be interpreted with caution.

In addition, statistical analyses were limited to chi-squared tests because the available datasets report aggregated categorical counts rather than individual-level data and do not include consistent denominators. As a result, analyses could assess differences in the distribution of deaths across age and gender categories but could not estimate mortality rates, relative risks, or other individual-level associations.

Fourth, age and gender categories were not standardized across sources, and data for gender diverse individuals were often limited by small sample sizes. Women and gender diverse people may also be underrepresented in publicly available data due to higher reliance on forms of ‘hidden homelessness’, such as couch surfing or temporary housing arrangements [14, 15, 47]. These inconsistencies and small counts limited direct comparison of age-specific shelter utilization and mortality patterns.

Finally, information on shelter operations and service availability was incomplete and often inconsistent. Service data were derived from publicly available shelter websites, many of which did not list basic services or hours of operation. These gaps conflict with City of Toronto shelter standards, which require 24/7 operation and provision of core services [68]. Consequently, the service availability analysis reflects publicly reported information rather than verified service delivery. Additionally, service counts represent the number of shelters offering a particular service, while percentages reflect the proportion within each shelter type (men-only, women-only, mixed-adult), meaning absolute counts and percentages may not directly align.

Together, these limitations highlight the need for standardized, transparent, and gender-inclusive data collection and reporting practices across homelessness and public health systems in Toronto. Without more comprehensive and consistent data, the full extent of gender-based inequities cannot be adequately assessed, limiting the ability to address these disparities through service improvements.

## Conclusion

In 2024 and 2025, men accounted for the majority of people experiencing homelessness in Toronto and represented the largest proportion of individuals living outdoors and using shelter settings. However, our findings demonstrate important gender disparities across the homelessness and overdose continuum. Women and gender diverse individuals have fewer shelter options than men, as women-only shelters represent a relatively small proportion of overall shelter capacity. At the same time, harm reduction services were least commonly available in women-only shelters compared with mixed-adult or men-only shelters.

Gender service gaps are particularly concerning given that overdose was the leading cause of death among women and gender-diverse individuals experiencing homelessness, with a higher proportion of women dying from overdose compared to men. Overdose deaths among women were also concentrated among younger age groups. Together, these findings highlight a misalignment between the distribution of shelter and harm reduction services and the populations experiencing the greatest burden of mortality.

Addressing persistent gendered service gaps will require investments in safe, accessible, and low-barrier supports for women and gender-diverse people experiencing homelessness. Expanding women-only and gender-diverse shelter spaces and increasing the availability of harm reduction services within these settings may help address the disparities in overdose mortality identified in this study. More broadly, strengthening trauma-informed and gender-responsive approaches within homelessness and harm reduction systems may improve access to care and reduce preventable deaths among populations facing disproportionate burden.

## Supplementary Information

Supplementary Material 1.

## Data Availability

The datasets generated and/or analysed during the current study are available in the following repositories: 1. *Demographics of People Experiencing Homelessness* and the *Demographic Profile of the Shelter Population* : [[https://www.toronto.ca/wp-content/uploads/2025/07/9790-street-needs-assessment-report-2024.pdf](https:/www.toronto.ca/wp-content/uploads/2025/07/9790-street-needs-assessment-report-2024.pdf)] 2. *Shelter Utilization* : [[https://open.toronto.ca/dataset/toronto-shelter-system-flow/](https:/open.toronto.ca/dataset/toronto-shelter-system-flow)] 3. *Deaths of Shelter Residents* : [ [https://www.toronto.ca/city-government/data-research-maps/research-reports/housing-and-homelessness-research-and-reports/deaths-of-shelter-residents/](https:/www.toronto.ca/city-government/data-research-maps/research-reports/housing-and-homelessness-research-and-reports/deaths-of-shelter-residents)] 4. *Shelter Capacity and Eligibility* : [[https://open.toronto.ca/dataset/daily-shelter-overnight-service-occupancy-capacity/](https:/open.toronto.ca/dataset/daily-shelter-overnight-service-occupancy-capacity) 5. *Deaths Among People Experiencing Homelessness in Toronto: * [[https://public.tableau.com/app/profile/tphseu/viz/DeathsofPeopleExperiencingHomelessness2_0/HomelessDeaths3_0](https:/public.tableau.com/app/profile/tphseu/viz/DeathsofPeopleExperiencingHomelessness2_0/HomelessDeaths3_0)] 6. *Shelter Service Provision and Availability: * see **Appendix A** for a list of websites used for data collection.

## Abbreviations

CAMH: Centre for Addiction and Mental Health
CRISM: Canadian Research Initiative in Substance Matters
SCS: Supervised Consumption Service
UKE: University Medical Center Hamburg-Eppendorf
VAW: Violence Against Women
ZIS: Center for Interdisciplinary Addiction Research
