# Supplementary material for: Dying younger, dying of overdose: gendered and age dimensions of mortality and shelter service access among individuals experiencing homelessness in Toronto, Canada
Source: BMC Public Health. 2026 Apr 22;26:1497. doi: 10.1186/s12889-026-27460-8 (PMC13151364; doi:10.1186/s12889-026-27460-8)
Supplement: Supplementary file 1 — Supplementary Material 1. [file 12889_2026_27460_MOESM1_ESM.docx]

**Appendix**

Appendix A

Definitions of Relevant Terms

| **Term** | **Definition** |
| --- | --- |
| Sex: | The classification of male, female, and intersex based on reproductive organs.^54^ |
| Gender: | The gender that people identify with. This may be different from their sex-assigned at birth.^54^ |
| Gender Diverse: | Individuals who identify as the following genders: transgender, non-binary, two-spirit, and/or gender fluid.^55^ |
| Homelessness: | Any individual who was either sleeping outdoors, staying in city-administered sites (such as shelters, triage programs, or respite sites), or in provincially-administered violence against women (VAW) facilities.^14^ |
| Individuals Experiencing Chronic Homelessness: | An individual who has experienced homelessness for at least six months.^14^ |
| Newly Identified: | Individuals entering the shelter system for the first time.^15^ |
| Returned from Housing: | Individuals who returned to the shelter after being marked as ‘moved to housing’.^15^ |
| Returned to Shelter: | Individuals who have returned to the shelter system after not accessing the shelter system for at least three months.^15^ |
| Mixed-Adults: | No gender specification for services.^56^ |
| Housing Supports: | Services which provide support in obtaining permanent housing and connections to transitional housing.^56^ |
| Acute Drug Toxicity | Referred to as an overdose |
| Hours of Operation | Hours of operation for shelter bed access. All Toronto shelters are open and staffed 24/7.^56^ |
| Bridging and Triage Programs | Hotel programs which provide temporary housing to individuals unable to access a space in the shelter.^14^ |
| Youth Shelter Services | Any shelter listed as‘youth’ on the *Daily Shelter & Overnight Service Occupancy & Capacity* dataset.^17^ |
| Trauma-Informed Care | Acknowledges that people experiencing homelessness often have past experiences of trauma which must be considered to ensure a safe, non-traumatizing environment.^57^ |
| Supervised Consumption Site (SCS) | A harm reduction service which provides a clean and supervised setting for people who use drugs to consume substances in the presence of trained staff.^58^ |

Appendix B

Toronto Shelters and their Associated Website

| **Shelter Program Name** | **Website** |
| --- | --- |
| **Men-Only** | |
| CONC Men's Hotel Program | <https://www.conccommunity.org/shelter-roncesvalles/> |
| Christie Ossington Men's Hostel | <https://www.conccommunity.org/shelter-housing-lansdowne/> |
| Christie Ossington Men's Hostel South | <https://www.conccommunity.org/shelter-bloor/> |
| Downsview Dells Bedded Program | <https://www.torontocentralhealthline.ca/displayservice.aspx?id=133835> |
| Fort York Residence Bedded Program | <https://www.torontocentralhealthline.ca/displayservice.aspx?id=133836> |
| Fort York SRO Units Program | <https://www.torontocentralhealthline.ca/displayservice.aspx?id=133836> |
| Progress Shelter | <https://211central.ca/record/78894266/> |
| Junction Place | <https://www.torontocentralhealthline.ca/displayservice.aspx?id=192448> |
| Seaton House - Annex Harm Reduction & Managed Alcohol Program | <https://www.torontocentralhealthline.ca/displayservice.aspx?id=132632> |
| Seaton House - Fourth Floor Program | <https://www.torontocentralhealthline.ca/displayservice.aspx?id=132632> |
| Seaton House - Infirmary Bedded Program | <https://www.torontocentralhealthline.ca/displayservice.aspx?id=132632> |
| Seaton House Hostel Response Program | <https://www.torontocentralhealthline.ca/displayservice.aspx?id=132632> |
| Cornerstone Place | <https://211central.ca/record/75496449/> |
| Dixon Hall - Schoolhouse | <https://www.centraleasthealthline.ca/displayservice.aspx?id=132846> |
| Good Shepherd - D.A.R.E. | <https://goodshepherd.ca/addiction-healthcare-services/> |
| Good Shepherd - Emergency/Resettlement | <https://goodshepherd.ca/shelter-support-services/> |
| Good Shepherd Barrett House - Men's Program | <https://goodshepherd.ca/supportive-housing/> |
| Good Shepherd Centre West End Hotel - Men's Program |  |
| 545 Lake Shore Blvd W. Men | <https://homesfirst.on.ca/hf_property/bathurst-lake-shore-shelter/> |
| 545 Lakeshore Blvd W. Men - Lower Level | <https://homesfirst.on.ca/hf_property/bathurst-lake-shore-shelter/> |
| HFS - Willowdale - Building A - Refugee Men | <https://homesfirst.on.ca/hf_property/willowdale-welcome-centre-shelter/> |
| Na-Me-Res (Native Men's Residence) | <https://www.nameres.org/housing/> |
| Na-Me-Res (Native Men's Residence) - Top Bunk | <https://www.nameres.org/housing/> |
| Sagatay | <https://www.nameres.org/housing/> |
| St. Simon's Clubbe House | <https://clubbehouse.com/> |
| Salvation Army - Gateway - Men's Hostel | <https://thegateway.ca/about/> |
| SA Scarborough Hotel - Men's Program | <https://torontohhs.org/shelters/temp-locations/> |
| Salvation Army - Maxwell Meighen - Men's Hostel | <https://torontohhs.org/shelters/maxwell-meighen/> |
| Salvation Army - New Hope - Hotel 400 - Men | <https://torontohhs.org/shelters/new-hope/> |
| Salvation Army - New Hope - Men's Hostel | <https://torontohhs.org/shelters/new-hope/> |
| Salvation Army - Islington Seniors' Shelter Men's Program | <https://torontohhs.org/shelters/islington-seniors-shelter/> |
| Scott Mission - Men's Ministry Overnight Program | <https://scottmission.com/programs/mens-ministry/> |
| Scott Mission - Respite | <https://scottmission.com/programs/mens-ministry/> |
| **Women-Only** | |
| Scarborough Women's Shelter - Milner | no website available |
| Women' s Residence - Main Program | <https://www.torontocentralhealthline.ca/displayservice.aspx?id=133394> |
| Fred Victor T2H Bedded Program | <https://www.fredvictor.org/what-we-do/housing/transitional-housing/> |
| Fred Victor Transition to Housing Bedded Program | <https://www.fredvictor.org/what-we-do/housing/transitional-housing/> |
| Fred Victor The Phoenix Women's 24/7 Drop-In | <https://www.fredvictor.org/what-we-do/health-services/drop-ins-community-programs/> |
| Fred Victor Women's Hostel Program | <https://www.fredvictor.org/what-we-do/housing/shelters/> |
| Homes First Society- Kennedy Shelter Women's Program | <https://homesfirst.on.ca/hf_property/kennedy-road-shelter/> |
| 545 Lake Shore Blvd W. Women | <https://homesfirst.on.ca/hf_property/bathurst-lake-shore-shelter/> |
| HFS - Willowdale - Building B - Refugee Women | <https://homesfirst.on.ca/hf_property/willowdale-welcome-centre-shelter/> |
| Sistering Overnight Women's Program | <https://sistering.org/low-barrier-drop-in/> |
| St.Vincent De Paul - Amelie House - Women's Shelter | <https://ssvptoronto.ca/residential-emergency-housing/> |
| Society of Saint Vincent de Paul - Elisa House - Top Bunk | <https://ssvptoronto.ca/residential-emergency-housing/> |
| St.Vincent De Paul - Elisa House | <https://ssvptoronto.ca/residential-emergency-housing/> |
| St.Vincent De Paul - Mary's Home - Emergency Shelter | <https://ssvptoronto.ca/residential-emergency-housing/> |
| St.Vincent De Paul - St. Clare's Residence - Transitional Housing | <https://ssvptoronto.ca/residential-emergency-housing/> |
| Street Haven - Emergency Hostel | <https://streethaven.org/services/emergency-shelter-services/> |
| Street Haven Hotel Program | no website available |
| Salvation Army - Evangeline Residence - Women's Ministry | <https://torontohhs.org/shelters/evangeline-residence/> |
| Salvation Army - Florence Booth - Hotel 400 - Women | <https://torontohhs.org/shelters/florence-booth/> |
| Salvation Army - Islington Seniors' Shelter Women's Program | <https://torontohhs.org/shelters/islington-seniors-shelter/> |
| Nellie's Women's Shelter | <https://nellies.org/what-we-do/shelter/> |
| YWCA - Adult Women Shelter | <https://www.ywcatoronto.org/ourprograms/shelterandhousing/emergencyshelterfromviolence/ywcawomensshelter> |
| YWCA Davenport - Adult | <https://www.ywcatoronto.org/ourprograms/shelterandhousing/homelessshelter/davenportshelter> |
| **Mixed-Adult** | |
| COSTI - Hotel Program - Dixon (Refugee Singles) | <https://www.costi.org/programs/program_details.php?sid=62&pid=13&id=222> |
| COSTI Reception Centre CITY Program | <https://www.costi.org/programs/program_details.php?sid=0&pid=0&id=164> |
| COSTI Reception Ctr CITY Program - Top Bunk | <https://www.costi.org/programs/program_details.php?sid=0&pid=0&id=164> |
| CMHA-TO - Carlton House - Mental Health & Addictions - Solace 2 | <https://cmhato.org/programs/carlton-house/> |
| CMHA-TO - Carlton House - Mental Health & Addictions - Solace 1 | <https://cmhato.org/programs/carlton-house/> |
| CONC Etobicoke Hotel Program - Mixed Adult | <https://www.conccommunity.org/shelter-rexdale/> |
| TSSS Etobicoke Hotel Program | <https://www.toronto.ca/city-government/accountability-operations-customer-service/city-administration/staff-directory-divisions-and-customer-service/toronto-shelter-support-services/> |
| Scarborough Village Residence Main Program | <https://www.torontocentralhealthline.ca/displayservice.aspx?id=51540> |
| Streets to Homes Bedded Program | <https://www.toronto.ca/community-people/housing-shelter/homeless-help/streets-to-homes-street-outreach-support-program/> |
| Fort York Residence North Hotel Program | <https://www.torontocentralhealthline.ca/displayservice.aspx?id=133836> |
| TSSS North York West Hotel - North - Couples | <https://www.toronto.ca/community-people/housing-shelter/homeless-help/about-torontos-shelter-system/developing-shelter-sites/1677-wilson-avenue/> |
| TSSS North York West Hotel - North - Mixed Adult | <https://www.toronto.ca/community-people/housing-shelter/homeless-help/about-torontos-shelter-system/developing-shelter-sites/1677-wilson-avenue/> |
| TSSS North York West Hotel - South - Couples | <https://www.toronto.ca/community-people/housing-shelter/homeless-help/about-torontos-shelter-system/developing-shelter-sites/1677-wilson-avenue/> |
| TSSS North York West Hotel Program - Mixed Adult | <https://www.toronto.ca/community-people/housing-shelter/homeless-help/about-torontos-shelter-system/developing-shelter-sites/1677-wilson-avenue/> |
| TSSS Downtown Hotel Women's Program | no website available |
| Dixon Hall 351 Lake Shore Blvd E. Respite | <https://dixonhall.org/housing-services/> |
| Dixon Hall - 354 George | <https://dixonhall.org/housing-services/> |
| Dixon Hall - Heyworth House | <https://dixonhall.org/housing-services/> |
| Fife House Denison Program | <https://www.fifehouse.org/programs-services/supportive-housing-programs/> |
| Fife-Sherbourne Transitional Program | <https://www.fifehouse.org/programs-services/supportive-housing-programs/> |
| Fred Victor Bethlehem United Program | <https://www.fredvictor.org/what-we-do/housing/shelters/> |
| Fred Victor Uptown Hotel Program | <https://www.fredvictor.org/what-we-do/housing/shelters/> |
| Fred Victor 1A Strachan Ave Respite | <https://www.fredvictor.org/what-we-do/housing/shelters/> |
| 545 Lake Shore Blvd W. Couples | <https://homesfirst.on.ca/hf_property/bathurst-lake-shore-shelter/> |
| HFS - Lawrence East Shelter | <https://homesfirst.on.ca/hf_property/4117-lawrence/> |
| HFS Scarborough Hotel Program - Mixed Adult | <https://homesfirst.on.ca/hf_property/delta-hotel-shelter/> |
| Homes First Society - Metro - Refugee Singles | <https://homesfirst.on.ca/hf_property/metro-shelter/> |
| Homes First Society - Pacewood | <https://homesfirst.on.ca/hf_property/pacewood/> |
| Homes First Society - Placer Mixed Adult Program | <https://homesfirst.on.ca/hf_property/101-placer/> |
| Homes First Society - Scarborough Shelter | <https://homesfirst.on.ca/hf_property/st-clair/> |
| La Passerelle I.D.E. - L'Agapanthe - Maison de Transition | no website found that matches the location |
| Margaret's 21 Park Rd. Respite | <https://margarets.ca/drop-in-services/> |
| St. Felix Centre 69 Fraser Respite | <https://stfelixcentre.org/programs-services/respite-services-program/> |
| St. Felix Centre - 629 Adelaide St. | <https://stfelixcentre.org/programs-services/respite-services-program/> |
| Sojourn House Bedded Program | <https://www.sojournhouse.org/programs-services/emergency-shelter/> |
| Toronto Community Hostel (Refugee Singles) | <https://www.centraleasthealthline.ca/displayservice.aspx?id=132827> |
| Warden Woods - Kingston Road Shelter | <https://wardenwoods.com/programs/shelter-services/> |
| **Youth** | |
| Turning Point Youth Services Shelter | <https://www.woodgreen.org/programs/emergency-youth-shelter> |
| YWCA - Youth Shelter | <https://www.ywcatoronto.org/ourprograms/shelterandhousing/homelessshelter/1ststopwoodlawnshelter> |
| YWCA Davenport - Youth | <https://www.ywcatoronto.org/ourprograms/shelterandhousing/homelessshelter/davenportshelter> |
| YMCA Sprott House | <https://www.ymcagta.org/youth-programs/youth-housing?_gl=1*97kmcj*_gcl_au*MTYyNjA3MzUyLjE3NTg4MDg1Mzk.*_ga*ODU0ODkzNzI2LjE3NTg4MDg1Mzk.*_ga_DYQQWTV52L*czE3NjQwMDA5MTgkbzMkZzAkdDE3NjQwMDA5MTgkajYwJGwwJGgxNDkzNDc2Mzc0> |
| Friends of Ruby - Transitional Housing | <https://www.friendsofruby.ca/transitional-housing/> |
| Centre for Refugee Children - Anchor House | <https://crcrefugee.ca/anchor-house/> |
| Covenant House Residence | <https://covenanthousetoronto.ca/our-solution/housing-and-aftercare/> |
| Covenant House Rights of Passage | <https://covenanthousetoronto.ca/our-solution/housing-and-aftercare/> |
| Eva's Phoenix | <https://www.evas.ca/where-we-are/evas-phoenix/> |
| Eva's Place | <https://www.evas.ca/where-we-are/evas-place/> |
| Eva's Satellite - Canterbury Place | <https://www.evas.ca/where-we-are/evas-satellite/> |
| Horizons for Youth | <https://horizonsforyouth.org/howwehelp> |
| Kennedy House Youth Shelter Bedded Program | <https://kennedyhouse.org/services/youth-shelter/> |
| Native Child & Family Services Toronto | <https://nativechild.org/youth/housing/> |
| Niizhoodenh Mshiikenhik - Twin Turtles | <https://nativechild.org/youth/housing/> |
| YMCA House-Vanauley | <https://www.ymcagta.org/youth-programs/youth-housing?_gl=1*97kmcj*_gcl_au*MTYyNjA3MzUyLjE3NTg4MDg1Mzk.*_ga*ODU0ODkzNzI2LjE3NTg4MDg1Mzk.*_ga_DYQQWTV52L*czE3NjQwMDA5MTgkbzMkZzAkdDE3NjQwMDA5MTgkajYwJGwwJGgxNDkzNDc2Mzc0> |
| Youth without Shelter Emergency Shelter Program | <https://yws.on.ca/how-we-help/emergency-residential/> |
| Youth without Shelter Stay In School Program | <https://yws.on.ca/how-we-help/stay-in-school/> |
| YouthLink Emergency Program | <https://youthlink.ca/services/our-programs/housing-and-shelter/youthlink-shelter/> |
| YouthLink Transitional Program | <https://youthlink.ca/services/our-programs/housing-and-shelter/transitional-housing-program/> |
| Covenant House Residence - Refugee Youths | program not listed on website |
| Friends of Ruby - Emergency Housing | program not listed on website |
| CHT Thrive Transitional shelter for refugee youth | program not listed on website |
| Covenant House - Transitional Safe Beds for women | program not listed on website |
| Covenant House - Madison House - Refugee Youth | program not listed on website |

Appendix C

Location and Age of Death Among People Experiencing Homelessness in 2024 Disaggregated by Gender

|  | **Outdoors** | | **Shelters** | | **Private Residences** | | **Hospitals/Clinics** | |
| --- | --- | --- | --- | --- | --- | --- | --- | --- |
|  | **n** | **%** | **n** | **%** | **n** | **%** | **n** | **%** |
| **Men** | | | | | | | | |
| **Under 20** | 0 | 0% | 1 | 3% | 0 | 0% | 0 | 0% |
| **20-39** | 14 | 27% | 9 | 31% | 8 | 28% | 5 | 22% |
| **40-59** | 32 | 62% | 15 | 52% | 12 | 42% | 10 | 43% |
| **60+** | 5 | 10% | 4 | 14% | 9 | 31% | 8 | 35% |
| **Unknown** | 1 | 2% | 0 | 0% | 0 | 0% | 0 | 0% |
| **Women** | | | | | | | | |
| **Under 20** | 0 | 0% | 1 | 10% | 0 | 0% | 0 | 0% |
| **20-39** | 8 | 53% | 3 | 30% | 7 | 88% | 1 | 20% |
| **40-59** | 6 | 40% | 3 | 30% | 1 | 13% | 1 | 20% |
| **60+** | 1 | 7% | 3 | 30% | 0 | 0% | 3 | 60% |
| **Unknown** | 0 | 0% | 0 | 0% | 0 | 0% | 0 | 0% |
| **Gender Diverse Individuals** | | | | | | | | |
| **Under 20** | 0 | 0% | 0 | 0% | 0 | 0% | 0 | 0% |
| **20-39** | 0 | 0% | 1 | 33% | 0 | 0% | 0 | 0% |
| **40-59** | 1 | 100% | 2 | 67% | 0 | 0% | 0 | 0% |
| **60+** | 0 | 0% | 0 | 0% | 0 | 0% | 1 | 100% |
| **Unknown** | 0 | 0% | 0 | 0% | 0 | 0% | 0 | 0% |

Appendix D

Table 1: Chi-Square Test for Outdoor Deaths by Age and Gender

|  | **Men** | **Women and Gender Diverse** | **Total** |
| --- | --- | --- | --- |
| **<40** | 14.00 (O)  38.69 (E) | 8.00 (O)  5.25 (E) | 22.00 |
| **≥40 years** | 37.00 (O)  34.25 (E) | 8.00 (O)  10.75 (E) | 45.00 |
| **Total** | 51.00 | 16.00 | 67.00 |
| X^2^ = 18.11316271  df = 1  p = 0.00002081575089 | | | |

Table 2: Chi-Square Test for Shelter Deaths by Age and Gender

|  | **Men** | **Women and Gender Diverse** | **Total** |
| --- | --- | --- | --- |
| **<40** | 10.00 (O)  15.00 (E) | 5.00 (O)  4.64 (E) | 15.00 |
| **≥40 years** | 19.00 (O)  18.64 (E) | 8.00 (O)  8.36 (E) | 27.00 |
| **Total** | 29.00 | 13.00 | 42.00 |
| X^2^ = 1.716243527  df = 1  p = 0.1901773045 | | | |

Table 3: Chi-Square Test for Overdose Deaths by Age and Gender

|  | **Men** | **Women and Gender Diverse** | **Total** |
| --- | --- | --- | --- |
| **<40** | 31.00 (O)  36.67 (E) | 24.00 (O)  18.33 (E) | 55.00 |
| **≥40 years** | 45.00 (O)  39.33 (E) | 14.00 (O)  19.67 (E) | 59.00 |
| **Total** | 76.00 | 38.00 | 114.00 |
| X^2^ = 5.07642527  df = 1  p = 0.02425332488 | | | |
